# Valproic acid reprograms the metabolic aberration of cisplatin treatment via ALDH modulation in triple-negative breast cancer cells

**DOI:** 10.3389/fcell.2023.1217149

**Published:** 2023-10-26

**Authors:** Avital Granit Mizrahi, Ahinoam Gugenheim, Haneen Hamad, Roa’a Hamed, Nino Tetro, Ofra Maimon, Salome Khutsurauli, Hovav Nechushtan, Benjamin Nisman, Deborah Duran, Widad Samman, Liron Birimberg-Schwartz, Myriam Grunewald, Sara Eyal, Tamar Peretz

**Affiliations:** ^1^ Oncology Laboratory, Sharett Institute of Oncology, Hadassah-Hebrew University Medical Center, Jerusalem, Israel; ^2^ School of Pharmacy, Institute for Drug Research, The Hebrew University, Jerusalem, Israel; ^3^ Faculty of Medicine, Hebrew University, Jerusalem, Israel; ^4^ Hadassah Organoid Center, The Hadassah Medical Organization, Jerusalem, Israel; ^5^ Department of Pediatric Gastroenterology, The Hadassah Medical Organization, Jerusalem, Israel

**Keywords:** valproic acid, cisplatin, disulfiram, glucose, metabolism, aldehyde dehydrogenase, ALDH, GLUT1

## Abstract

We recently demonstrated that the histone deacetylase inhibitor valproic acid (VPA) reprograms the cisplatin-induced metabolome of triple-negative breast cancer (TNBC) cells, including a shift in hexose levels. Accordingly, here, we tested the hypothesis that VPA alters glucose metabolism in correlation with cisplatin sensitivity. Two TNBC cell lines, MDA-MB-231 (a cisplatin-resistant line) and MDA-MB-436 (a cisplatin-sensitive line), were analyzed. The glycolysis and oxidative metabolism were measured using the Glycolysis Stress Test kit. The expression of aldehyde dehydrogenases (ALDHs), enzymes linked to drug resistance, was investigated by Western blot and real-time PCR analyses. We additionally studied the influence of ALDH inhibition by disulfiram on the viability of MDA-MB-231 cells and on a TNBC patient-derived organoid system. Cisplatin treatment reduced the extracellular acidification rate in MDA-MB-436 cells but not MDA-MB-231 cells, whereas VPA addition increased the extracellular acidification rate in both cell lines. VPA further reduced the oxygen consumption rate of cisplatin-treated MDA-MB-436 cells, which correlated with cell cycle alterations. However, in MDA-MB-231 cells, the cell cycle distribution did not change between cisplatin/VPA–cisplatin treatments. In both cell lines, VPA increased the expression of ALDH isoform and ALDH1A1 expression. However, only in MDA-MB-231 cells, VPA synergized with cisplatin to augment this effect. Disulfiram sensitized the cells to the cytotoxic effects of the VPA–cisplatin combination. Furthermore, the disulfiram–VPA–chemotherapy combination was most effective in TNBC organoids. Our results show that ALDH overexpression may act as one mechanism of cellular resistance to VPA in TNBC and that its inhibition may enhance the therapeutic efficacy of VPA–chemotherapeutic drug combinations.

## Introduction

Triple-negative breast cancer (TNBC) represents 15% of breast carcinomas and is defined by the absence of the three main breast cancer biomarkers—estrogen receptors, progesterone receptors, and HER2 (also known as ERBB2) (Denkert et al., 2017). Treatment regimens include taxanes, anthracyclines, platinum compounds, topoisomerase inhibitors (e.g., topotecan), and therapeutic proteins (Marra et al., 2020; Loibl et al., 2021). Despite advances in pharmacotherapy, TNBC has relatively poor outcomes with a peak risk of disease recurrence at ∼3 years after treatment (von Minckwitz et al., 2012). Among the causes of treatment failure are altered drug uptake, drug efflux, remodeling of DNA repair pathways, and metabolic reprograming (Ranasinghe et al., 2022).

Metabolic reprogramming is a key characteristic of cancer cells that distinguishes them from normal cells. One feature is the Warburg effect, in which tumor cells produce energy through high rate of glycolysis rather than mitochondrial oxidative phosphorylation (OXPHOS), even in the presence of oxygen (Warburg, 1956). The altered glucose metabolism contributes to cancer progression and metastasis and is being utilized in diagnostic imaging with [^18^F]fluorodeoxyglucose ([^18^F]FDG) (Mankoff et al., 2007).

Innovative treatments of TNBC include histone deacetylase (HDAC) inhibitors, which have shown efficacy against TNBC in combination with chemotherapeutic agents (Luu et al., 2008; Yardley et al., 2013; Bilen et al., 2015; Wawruszak et al., 2021). Recently, we have shown that exposure of MDA-MD-231 cells to the HDAC inhibitors, valproic acid (VPA), generates profound changes in their metabolites profile and shifted the cisplatin-induced metabolic profile to higher levels of hexose and phosphatidylcholine, indicative of alteration in glucose and lipid metabolism (Granit et al., 2022). VPA-induced metabolic reprogramming has previously been observed in breast cancer stem cells (Debeb et al., 2016) and in a Fanconi anemia cell model (Bertola et al., 2023). This phenomenon has been linked to the ability of VPA to increase the activity of aldehyde dehydrogenase (ALDH). HDAC inhibitors have demonstrated the ability to epigenetically modify the ALDH isotype, specifically the ALDH1A1 expression, via interaction with the bromodomain and extraterminal (BET) family of proteins, which recognize acetylated lysine on histones through their bromodomains (Yokoyama et al., 2016). ALDHs may play an important role in the chemo-resistance ability, clonogenicity, and spherogenesis of the cancer stem cell (Ginestier et al., 2007; Wang et al., 2020), and the ALDH1A1 expression was correlated with poorer overall survival in breast cancer patients (Morimoto et al., 2009). In addition, ALDH overexpression was associated with poor prognostic features, including an increased tumor grade, extensive lymph node metastasis, and a greater extent of luminal B and triple-negative subtypes of breast cancer (Althobiti et al., 2020). The precise mechanism through which ALDHs regulate stemness remains partially understood. ALDH1A1 has also been shown to exhibit metabolic activity and contribute to the promotion of DNA repair, thereby affecting cancer progression. (Yue et al., 2022).

ALDH enzymes are involved in the detoxification of aldehydes in an NAD(P)+-dependent manner, thus reducing oxidative stress (Pors and Moreb, 2014). Chemotherapeutics and radiation therapy induce heightened levels of reactive oxygen species (ROS), resulting in oxidative stress within cancer cells, which contributes to their therapeutic efficacy (Marullo et al., 2013). Elevated ALDH expression potentially serves as a safeguard for cancer cells against these treatments, by maintaining ROS at low levels (Lawenda et al., 2008; Singh et al., 2013). In breast cancer cells, knockdown of ALDH1A1 increased the sensitivity to chemotherapy and radiotherapy (Croker et al., 2017). Additionally, VPA-treated breast cancer stem cells with ALDH activity are shown to be more resistant to chemotherapy (Debeb et al., 2012). ALDHs also participate in retinoic acid (RA) synthesis and can modulate the binding of the transcription factors retinoic acid receptor α (RAR), retinoic X receptor (RXR), and estrogen receptor α (ERα) to DNA, thus promoting cell proliferation, drug resistance, and inhibition of apoptosis (Zanoni et al., 2022).

In the current study, we focused on the effects of the following treatment on glucose metabolism of TNBC cells. Our aims were 1) assessing the effects of VPA, cisplatin, and their combination on glucose metabolism; 2) evaluating the expression of ALDH isoforms upon cisplatin and VPA treatment; and 3) evaluating the potential of the non-specific ALDH inhibitor disulfiram, which inhibits ALDHs, ALDH1A1, and ALDH2 (Koppaka et al., 2012), to reverse untoward effects of VPA–cisplatin. To address these aims, we used representative TNBC cell lines, cisplatin-sensitive cells (MDA-MB-436) and cells intrinsically resistant to cisplatin (MDA-MB-231) (Dominguez-Gomez et al., 2015; Dunne et al., 2018), and a TNBC patient-derived organoid model.

## Materials and methods

Sodium valproate, propidium iodide (PI), and red blood cell lysis buffer were purchased from Merck (KGaA, Darmstadt, Germany). Cisplatin was from Pharmachemie B.V (Haarlem, Netherlands). Disulfiram and the fluorescent 2-deoxyglucose analog 2-[N-(7-nitrobenz-2-oxa-1,3-diazol-4-yl)amino]-2-deoxy-D-glucose (2-NBDG) were from Cayman chemical (MI, USA). Paclitaxel was from Teva (Tel Aviv, Israel). GFR Matrigel was purchased from Corning (AZ, USA). All cell culturing reagents and the 2,3-bis(2-methoxy-4-nitro-5-sulfophenyl)-2H-tetrazolium-5-carboxanilide (XTT) Assay Kit were from Sartorius (Biological Industries Ltd., Beit Haemek, Israel). The Protease Inhibitor Cocktail Kit, the High-Capacity cDNA Reverse Transcription Kit, and RnaseA were purchased from Thermo Fisher Scientific (Waltham, MA, USA). The RNeasy Mini-Isolation Kit was from QIAGEN (Hilden, Germany). Xpert Fast SYBR was from Grisp (Porto, Portugal). Anti-ALDH1A1 was from R&D Systems (Minneapolis, MN, USA). Anti-H4, anti-AcH4, and anti-β-actin were purchased from Abcam (Cambridge, UK). The horseradish peroxidase (HRP)-conjugated goat anti-mouse secondary antibody was from Jackson ImmunoResearch (West Grove, PA, USA). The Bicinchoninic Acid (BCA) Protein Assay Kit was from Pierce (Rockford, IL, USA). The Glycolysis Stress Test kit was from Agilent (Santa Clara, CA, USA).

### Cell lines and cell culture

The MDA-MB-436 cells were from the American Type Culture Collection. The MDA-MB-231 cells were kindly provided by Prof. Michael Elkin (Hadassah Medical Center) and maintained in Dulbecco’s Modified Eagle Medium (DMEM) supplemented with 10% fetal calf serum, 1% penicillin, 1% streptomycin, and 1% glutamine. The cells were maintained at 37°C in an atmosphere of 5% CO_2_. For assessing treatment effects, they were incubated in a culture medium with 1 mM VPA (representing plasma concentrations in the order of magnitude which has been achieved in patients with solid tumors) (Atmaca et al., 2007), 10 μM cisplatin, 20 μM disulfiram, their combination, or the vehicle (0.1% DMSO).

### TBNC-derived organoid culture

The patient’s pleural effusion was obtained during thoracic drainage after obtaining written informed consent according to the protocol (#HMO-0921-20) approved by the Hadassah Medical Organization ethics committee and the Israeli Ministry of Health. The pleural effusion was collected in a 50 mL sterile tube, transported to the laboratory on ice and processed within 30 min as previously reported (Pan et al., 2021). In brief, the pleural effusion was strained through a 100 μm cell strainer and centrifuged at 250 g for 5 min. Red blood cells were lysed with red blood cell lysis buffer and washed with advanced DMEM-F12. The cell pellet was resuspended in GFR Matrigel (1.6 × 10^6^ cells/mL). Cell suspension droplets were deposited on a pre-heated 24-well culture plate which was inverted and placed at 37°C for 30 min to allow gelation. Then, 500 μl of complete medium (Advanced DMEM-F12, 1% penicillin, 1% streptomycin, 1% HEPES 1 M solution, 1% glutamine, 1 x B27, 5 ng/mL human neuregulin-1, 1.25 mM *N*-acetyl-L-cysteine, 5 mM nicotinamide, 5 ng/mL recombinant human epidermal growth factor (EGF), 20 ng/mL recombinant human fibroblast growth factor (FGF)-1, 5 ng/mL recombinant human FGF-7, 100 ng/mL recombinant human Noggin, 250 ng/mL recombinant human r-spondin-1, 500 nM SB202190, 10 μM Y-27632, 20 ng/mL human insulin-like growth factor-I, 10 nM 17β-estradiol, 50 nM hydrocortisone, and 1 x insulin-transferrin-selenium) was added in each well. Three-dimensional organoids were typically formed during the first 4 days of culture in a 37°C, 5% CO_2_ incubator. The medium was refreshed every 5 days, and confluent cultures were passaged at a ratio of 1–2 by mechanical disruption.

### Metabolic assays

Ten thousand cells were seeded per well in XF 96-well microplates and incubated for 24 h with the medium and then for an additional 72 h with the aforementioned treatments. Basal oxygen consumption rate (OCR) and extracellular acidification rate (ECAR) measurements were performed by the Seahorse XFe96 Analyzer (Agilent Technologies Inc., Santa Clara, CA, USA) using the Glycolysis Stress Test kit. Following completion of the measurements, cell viability was analyzed using the sodium 3′-[1-[(phenylamino)-carbony]-3,4-tetrazolium]-bis(4-methoxy-6-nitro)benzene-sulfonic acid hydrate (XTT) assay.

### Cell cycle analysis

For fluorescence-activated cell sorting (FACS) analysis, the cells were fixed overnight at 4°C in 70% ethanol and stained with PI for 1 h. The cells were analyzed using the CytoFLEX Platform flow cytometer (Beckman Coulter Life Sciences, Indianapolis, IN, USA).

### Immunoblotting

Approximately 10 million cells were harvested by trypsinization, washed in ice-cold phosphate-buffered saline (PBS), and lysed in 1 x radioimmunoprecipitation assay (RIPA) lysis buffer with the Halt™ Protease Inhibitor Cocktail Kit. Protein concentrations were determined by the BCA Protein Assay Kit. Thirty microgram protein underwent electrophoresis on 15% gradient sodium dodecyl sulfate (SDS)-polyacrylamide gels. Membranes were incubated with primary antibodies—anti-ALDH1A1 (1:450), anti-acetyl-H4 (1:10,000), anti-H4 (1:1,000), or anti-β-actin (1:1,000)—overnight at 4°C. The blots were then incubated for 1 h with a HRP-conjugated goat anti-mouse secondary antibody (1:5,000) and developed by enhanced chemiluminescence.

### Analyses of mRNA expression

Total RNA was isolated from one million cells using the RNeasy Mini-Isolation Kit according to the manufacturer’s instructions. The cDNA was synthesized by using the High-Capacity cDNA Reverse Transcription Kit in a 20 μL reaction containing 1 μg of total RNA. An aliquot of 1 μL cDNA was used in each 10 μL PCR reaction, using Xpert Fast SYBR, and reactions were run on an ABI StepOnePlus PCR system (Thermo Fisher Scientific). The primers used for PCR are listed in Supplementary Table S1.

### Viability assays

Cell and organoid viability were analyzed using the XTT Assay Kit, according to the manufacturer’s instructions. In brief, 10,000 cells were seeded in 96-well plates at 37°C, incubated for 24 h, and treated with the indicated concentrations of cisplatin alone or with 1 mM VPA, 20 µM disulfiram, or their combinations for 72 h. Organoids were harvested, washed, and resuspended in a complete medium. About 100 organoids were transferred to 96 wells that were pre-coated with a layer of 35 µl of Matrigel. Organoids were allowed to settle down on the Matrigel overnight in a 37°C, 5% CO_2_ incubator. Paclitaxel alone (7 µM) or with 1 mM VPA, 20 µM disulfiram, or their combinations were added to the growth medium. Organoids were exposed to the drug combinations for 5 days. To quantify the drug effect on cell or organoid viability, 50 µl of the XTT reagent was added to the medium, and the cells were further incubated for 2–4 h. The plate was analyzed by using a plate reader (Sunrise™, Tecan, Männedorf, Switzerland) at 450 nm.

### Uptake assays

Treated cells (1*10^6^) were cultured for 72 h and then washed with a glucose-free medium. The medium was replaced with glucose-free DMEM, and the cells were incubated for 1 hr at 37°C. 2-NBDG was added to the cells in glucose-free DMEM (final concentration 10 µM), and the cells were incubated for an additional 2 h at 37°C. The incubation medium was then removed, and the cells were washed twice with cold PBS. The cells were then trypsinized and resuspended in cold PBS with 4′,6-diamidino-2-phenylindole (DAPI; final concentration 1 μg/mL). The plate was read using the CytoFLEX Platform flow cytometer (Beckman Coulter Life Sciences, Indianapolis, IN, USA) at 525/540 nm.

### Data analysis

The OCR and ECAR values were normalized by the results of the XTT viability analysis. The flow cytometry data (10,000 cells/assay) were analyzed using the CytExpert software (Beckman Coulter). ImageLab (ChemiDocTMXRS+, Bio-Rad, USA) was used to quantify the densities of the target bands obtained by Western blotting. The results are expressed as the relative intensity ratio of the ALDH1A1 and β-actin bands or the relative intensity of AchH4 and H4 bands. The RT-PCR data were analyzed using StepOnePlus software v2.3 (Thermo Fisher Scientific, Waltham, MA, USA). Relative cell viability was calculated with the values of vehicle-treated cells set as 100%. Unless otherwise stated, studies were conducted in triplicate, on two different days (*n* = 6 per treatment group).

### Statistical analysis

The Kruskal–Wallis test followed by Dunn’s post-test was used to determine the statistical significance of the differences between experimental groups. Data are presented as mean and standard deviation. A *p*-value <0.05 was considered significant. The final inhibitory concentration (IC)_50_ value was determined by using the dose-response data with a linear regression model. Statistical analyses were performed using GraphPad Prism, version 8 (San Diego, CA, USA).

## Results

### VPA shifts the effects of cisplatin on the energy metabolism of breast cancer cells

**FIGURE 1:**
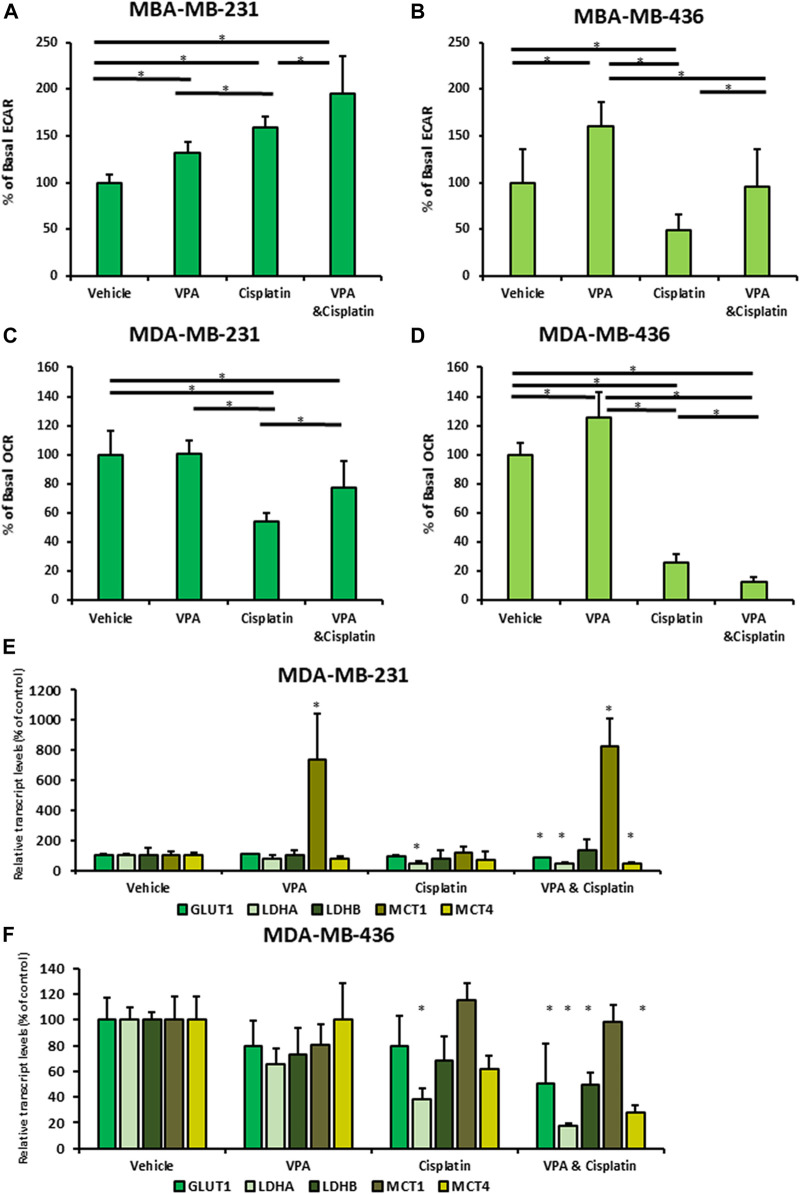
Vaplroic acid (VPA) effect on glucose and mitochondrial metabolism in MDA-MB-231 and MDA-MB-436 cells in the presence of cisplatin. MDA-MB-231 and MBA-MB-436 cells were exposed for 72 h to 1 mM VPA, 5 µM cisplatin (for MDA-MB-436), 10 µM cisplatin (for MDA-MB-231), or their combination. The cells were evaluated using a Seahorse XFe Extracellular Flux Analyzer. **(A,B)** Basal ECAR assessment of the glycolytic capacity of MBA-MB-231 **(A)** and MDA-MB-436 cells **(B)**. **(C,D)** OCR assessment of basal respiration for MDA-MB-231 **(C)** and MDA-MB-436 cells **(D)**. Results are presented as the percent of control (mean ± standard deviation [SD]); N = 5/group. **(E)** Real-time PCR of GLUT1, LDH A, LDH B, MCT1, and MCT4. MDA-MB-231 cells were treated with VPA, cisplatin, or both, for 72 h. **(F)** The same as mentioned above for MDA-MB-436 cells. Results are presented as the percent of control (mean ± standard deviation [SD]); *p < 0.05; N = 6/group.

For the ECAR analysis, cisplatin served as a positive control because cisplatin-resistant tumor cell lines are characterized by enhanced glycolysis (Qian et al., 2017). Cisplatin and VPA each increased the basal ECAR in MDA-MB-231 cells (cisplatin resistant; 1.6-fold and 1.3-fold, respectively; *p* < 0.05; Figure 1A) indicating higher glycolysis rate. The VPA–cisplatin combination further increased this effect (1.95-fold as compared to control, *p* < 0.05). Similarly, VPA increased the ECAR levels by 1.6-fold in MDA-MB-436 cells (cisplatin-sensitive; *p* < 0.05). However, in these cells, cisplatin reduced the ECAR level by one half (*p* < 0.05; Figure 1B), and VPA rescued the cells from the cisplatin effect. In MDA-MB-231 cells, cisplatin reduced the basal OCR level (indicating mitochondrial respiration) by half (*p* < 0.05), whereas VPA did not significantly affect it and partially rescued the cells from the cisplatin effect (Figure 1C). A 70% decrease (*p* < 0.05) in the basal OCR level was detected in MDA-MB-436 cells following cisplatin treatment. In contrast to MDA-MB-231 cells, VPA increased it by 1.25-fold (*p* < 0.05) as compared to control (Figure 1D). The VPA–cisplatin combination further decreased the OCR level (*p* < 0.05) as compared to cisplatin alone.

Next, to investigate the mechanism of the energy metabolism after VPA–cisplatin treatment, we examined the mRNA expression of glucose transporter 1 (GLUT1), lactate dehydrogenase (LDH) A and B, and monocarboxylate transporters 1 and 4 (MCT 1 and 4). The level of GLUT1, LDH A, and MCT4 mRNA expression was decreased after VPA–cisplatin treatment in both cell lines (Figures 1E, F). MCT1 levels were significantly higher in the presence of VPA in MDA-MB-231 cells but not in MDA-MB-436 cells.

### VPA affects cell cycle distribution in cisplatin-treated cells

**FIGURE 2:**
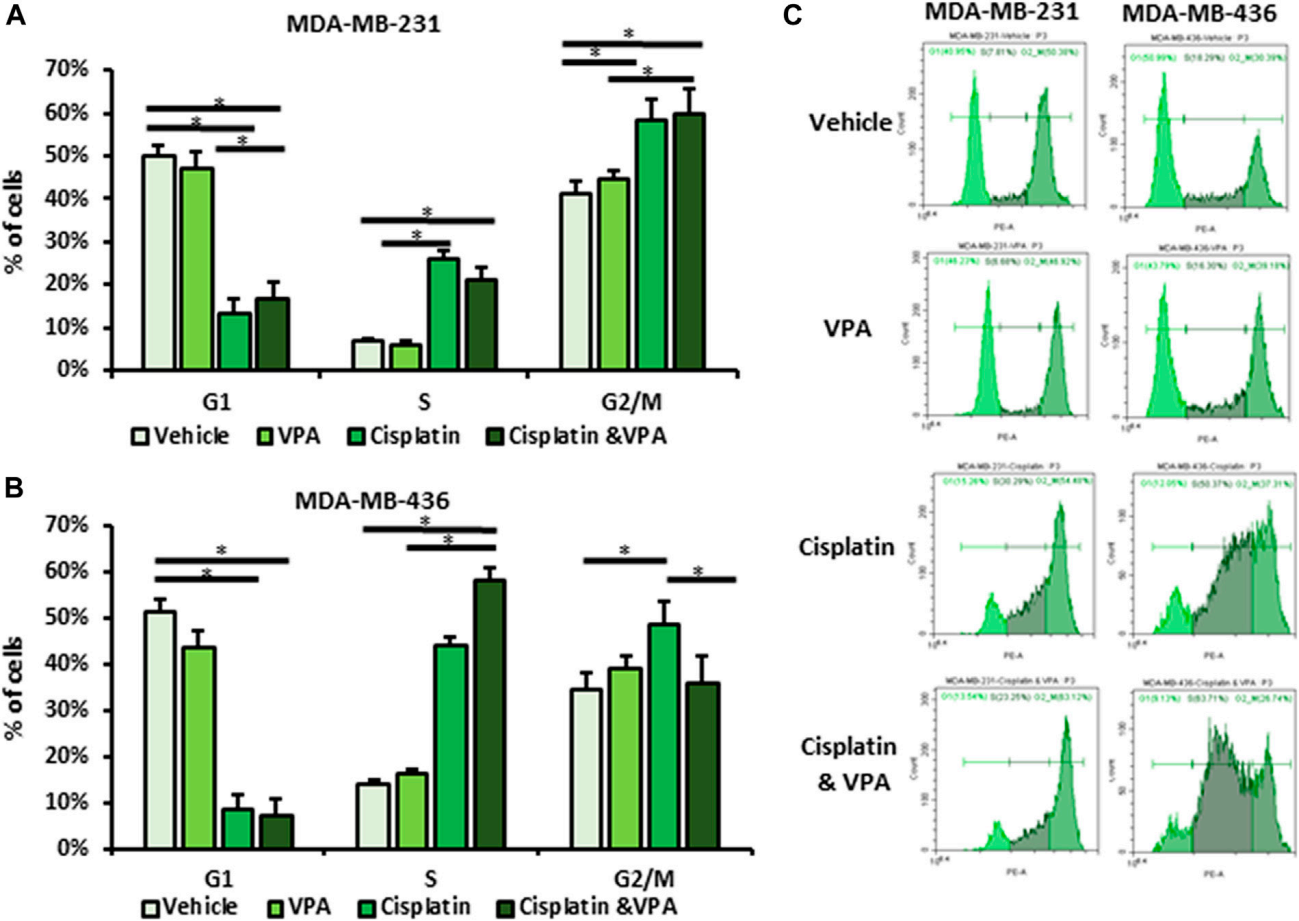
Effect of Valproic acid (VPA), cisplatin, and their combination on cell cycle distribution of MDA-MB-231 and MDA-MB-436 cells. The cells were treated with 1 mM of VPA, 5 µM cisplatin (for MDA-MB-436), 10 µM cisplatin (for MDA-MB-231), or their combination for 72 h. The cell cycle phases were analyzed by flow cytometry and propidium iodide (PI) labeling. **(A,B)** Cell cycle analysis of MBA-MB-231 **(A)** and MBA-MB-436 cells **(B)**. Results are presented as the percent of control (mean ± standard deviation [SD]); *p < 0.05; N = 6/group. **(C)** Representative flow cytometry histograms.

To further understand the phenotype of the metabolism shift in VPA–cisplatin-treated cells, we studied their cell cycle distribution using flow cytometry. In both cell lines, treatment with cisplatin resulted in increased proportion of the cells in the S and G2/M phases (Figures 2A–C). VPA-treated cells exhibited the same cell cycle distribution as the vehicle-treated controls. The addition of VPA to cisplatin did not alter the effect of cisplatin in MDA-MB-231 cells, but in MDA-MB-436 cells, the VPA–cisplatin treatment reduced the percentage of cells in the G2/M phase and increased early cell cycle arrest at the S phase by approximately 14% (*p* < 0.05) as compared to cisplatin-treated cells.

### VPA–cisplatin combination increases ALDH1A1 expression in an additive manner

**FIGURE 3:**
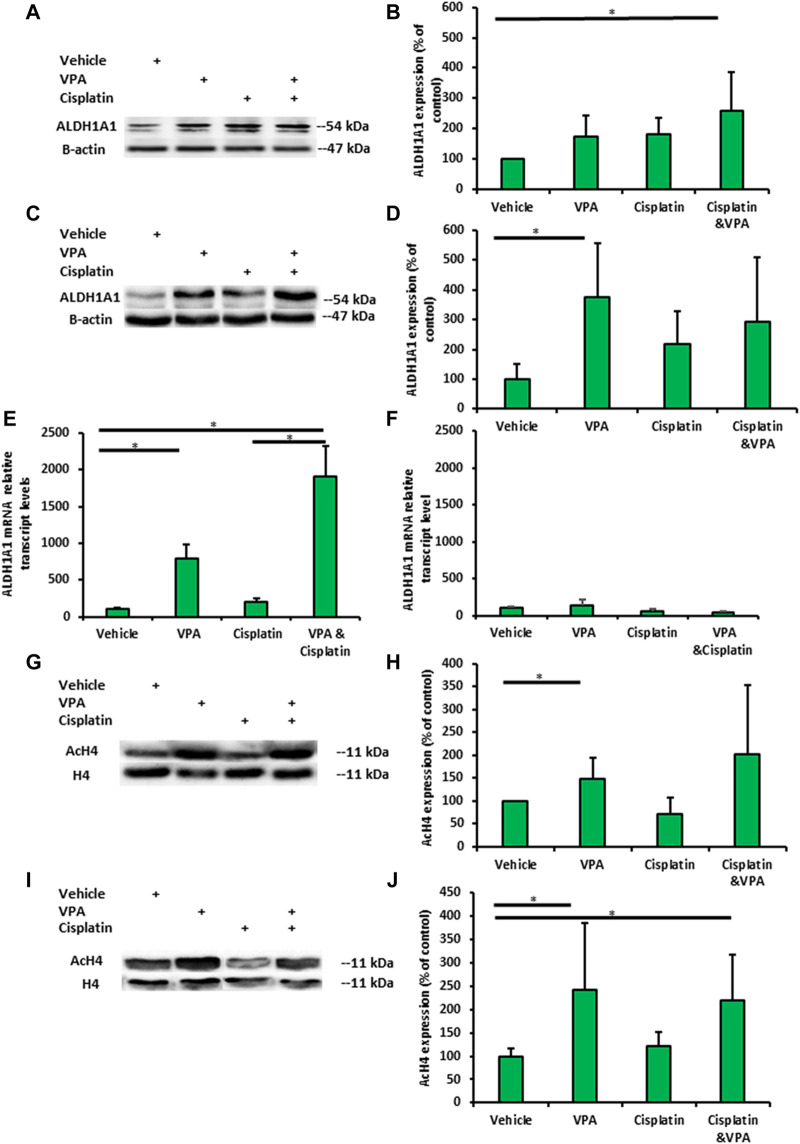
Valproic acid (VPA) and cisplatin exert an additive effect on ALDH1A1 expression in MDA-MB-231 cells but not on MDA-MB-436 cells. The cells were incubated with 1 mM VPA, 5 µM cisplatin (for MDA-MB-436), 10 µM cisplatin (MDA-NB-231), or their combination for 72 h. **(A)** Representative images of ALDH1A1 protein expression in MDA-MB-231 cells by Western blotting. **(B)** Quantification of the optic density. Results are presented as the percent of control (mean ± standard deviation [SD]); N = 6/group. **(C,D)** The same as mentioned above for MDA-MB-436 cells. **(E)** ALDH1A1 mRNA expression in MDA-MB-231 cells (by RT-PCR). Results are presented as the percent of control (mean ± standard deviation [SD]); N = 6/group. **(F)** The same as mentioned above for MDA-MB-436 cells. **(G)** Representative images of histone 4 acetylation in MDA-MB-231 cells. **(H)** Quantification of the optic density. Results are presented as the percent of control (mean ± standard deviation [SD]); N = 6/group. **(I,J)** The same as mentioned above for MDA-MB-436 cells. *p < 0.05.

We next addressed the question of whether VPA, cisplatin, and their combination affect the expression of the representative ALDH and ALDH1A1 in TNBC cells. Western blot analysis showed that ALDH1A1 was expressed at significantly higher levels in VPA–cisplatin-treated MDA-MB-231 cells and in VPA-treated MDA-MB-436 cells (Figures 3A–D). An analysis confirmed the effect of the drug combination on the ALDH1A1 expression also at the mRNA level in MDA-MB-231 cells (but not MDA-MB-436 cells). It additionally demonstrated a significant increase in ALDH1A1 transcript levels following treatment with VPA only (Figures 3E, F). However, only in the presence of cisplatin, higher ROS level was detected, and VPA addition did not modulate their amount (Supplementary Figure S2).

Next, we examined whether the H4 acetylation of MDA-MB-231- and MDA-MB-436-treated cells is associated with ALDH1A1 expression. The H4 acetylation levels VPA-treated MDA-MB-231 and MDA-MB-436 cells exhibited significant change in the H4 acetylation level. In addition, H4 acetylation was enhanced in MDA-MB-436 cells following treatment with VPA and VPA–cisplatin (*p* < 0.05) (Figures 3G–J).

### ALDH inhibition by disulfiram sensitizes MDA-MB-231 cells to VPA-cisplatin treatment

**FIGURE 4:**
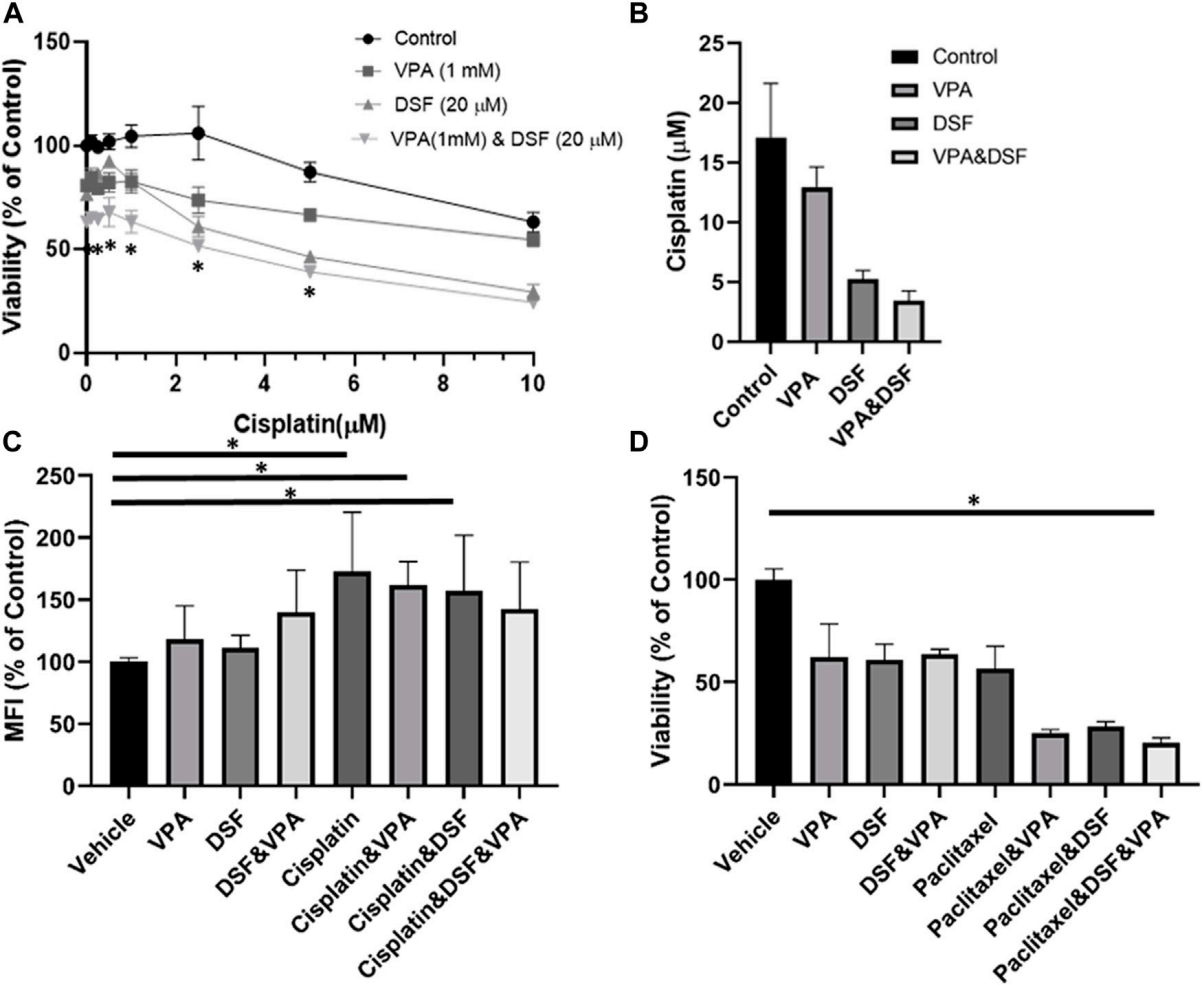
Disulfiram sensitizes MDA-MB-231 cells to the effects of VPA and chemotherapy. **(A)** MDA-MB-231 cells were treated with increasing concentration of cisplatin alone or with 1 mM Valproic acid (VPA), 20 µM disulfiram (DSF), or their combination for 72 h, followed by the XTT analysis of cellular viability. *p < 0.05 **(B)** Half-maximal inhibitory concentration (IC50) plotted from the cell viability analysis. **(C)** 2-NBDG uptake by MDA-MB-231 treated for 72 h with 1 mM VPA, 20 µM DSF, 10 μM of cisplatin, or their combination. 2-NBDG cellular accumulation was analyzed by flow cytometry. Results are presented as the percent of control (mean ± standard deviation [SD]); N = 6/group. **(D)** Effect of disulfiram on cell viability in VPA- and paclitaxel-treated organoids. TNBC pleural effusion-derived tumor organoids were treated with 1 mM VPA, 7 μM paclitaxel, 20 µM DSF, or their combination for 5 days, followed by the XTT assay for cellular viability. Results are presented as the percent of control (mean ± standard deviation [SD]); N = 4/group. *p < 0.05.

We sought to investigate whether non-specific ALDH inhibition can enhance the sensitivity of MDA-MB-231 to cisplatin and VPA treatment. Disulfiram, an anti-alcoholism medication, is an irreversible inhibitor of ALDH (Koppaka et al., 2012) with anti-cancer effect (Nechushtan et al., 2015). Indeed, the addition of disulfiram to the other treatments enhanced their cytotoxicity, producing the largest reduction in cell viability (Figure 4A). The IC50 value of cisplatin alone was 17.1 ± 4.5 µM. The addition of VPA, disulfiram, and a VPA–disulfiram combination to cisplatin reduced the IC50 values to 12.9 ± 1.7, 5.3 ± 0.7, and 3.5 ± 0.8 µM, respectively (Figure 4B).

We also measured the uptake of 2-NBDG, a fluorescent tracer used for monitoring glucose uptake into live cells. Disulfiram and VPA treatments did not affect the cellular uptake of 2-NBDG. As predicted, the uptake was 1.7-fold higher in the cisplatin-treated cells as compared to controls (Figure 4C) and remained elevated when cisplatin was combined with VPA or disulfiram. Interestingly, the disulfiram–VPA–cisplatin combination did not increase the 2-NBDG uptake in comparison with the control, suggesting an effect on glucose metabolism.

Finally, we examined the cytotoxic effect of the disulfiram–VPA–chemotherapy combination on pleural effusion-derived organoids from TNBC patients. Because cisplatin had no effect on cell viability (Supplementary Figure S3), we continued with another ROS inducing chemotherapy, paclitaxel (Samanta et al., 2014). In correlation with the results obtained for the MDA-MB-231 cells, the addition of disulfiram reduced the viability of VPA-chemotherapy-treated organoids (Figure 4D).

## Discussion

In recent years, there has been a growing interest in cancer metabolism and the impact of anti-cancer therapies on glucose utilization. This study aimed to characterize the metabolic response of TNBC cells to the addition of VPA to chemotherapy and identify its association with their sensitivity to treatment. A different response to VPA–cisplatin combination has been observed in breast cancer cell lines, and MDA-MB-231 cells were particularly resistant to this combination (Wawruszak et al., 2015). In addition, different responses of TNBC cells to drugs have been shown in metabolic rates and principle metabolic components, demonstrating the ability MDA-MB-231 cells to undergo metabolic adaption (Lanning et al., 2017). These findings are consistent with our previous metabolomics analyses, in which MDA-MB-231 cells also exhibited alterations in their metabolite profile after VPA–cisplatin treatment (Granit et al., 2022). Therefore, the different metabolic response between the TNBC cell lines in our present work extends these observations. Our findings not only revealed common patterns between TNBC cells but also identified cell line-specific responses. VPA addition to cisplatin treatment had a similar trend in the glycolysis pathway, increasing the ECAR in both cell lines compared to cisplatin alone (Figures 1A, B). In contrast to the reduced GLUT1, LDH A, and MCT4 transcript expression, MCT1 expression increased in MDA-MB-231 cells (Figures 1E, F). Furthermore, a diverse response was observed in the oxidative phosphorylation pathway represented by the OCR. VPA combined with cisplatin increased the OCR compared to cisplatin only in MDA-MB-231 cells but had the opposite effect in MDA-MB-436 cells (Figures 1C, D). MDA-MB-436 cells also exhibit early-phase cell cycle arrest, indicating an increased sensitivity to the VPA–cisplatin combination (Figure 2B). The difference in ECAR and OCR response to the VPA–cisplatin combination, together with the cell cycle alterations, help understand the common pathways and differences between these TNBC cell lines in a wider metabolic context. For example, our previous work demonstrated that the VPA–cisplatin combination increased the hexose level in MDA-MB-231 cells. Here, we showed a correlation with the ECAR for that observation. In addition, the metabolomic analysis assumed that VPA increased cisplatin sensitivity, which was the case for MDA-MB-436 but not MDA-MB-231 cells. Therefore, metabolite analysis combined with metabolic rates and cell cycle analysis can provide a deeper conception of drug-induced changes in metabolic profiles of TNBC cells.

VPA has been described as a potential anti-breast cancer treatment as monotherapy or combined with chemotherapy, such as cisplatin (Wawruszak et al., 2021). Cisplatin can cause DNA damage and metabolic reprogramming by inducing oxidative stress, as demonstrated for head and neck squamous cell carcinoma (HNSCC) (Yu et al., 2018). One mechanism by which tumor cells survive cisplatin treatment is the overexpression of ALDHs. ALDHs mediate the oxidation of a wide range of aldehydes to acids (Koppaka et al., 2012), and they are closely associated to drug resistance during conventional cancer chemotherapy (Liu et al., 2021). In breast cancer cell lines, ALDH activity also altered the metabolic phenotype by increasing the oxidative phosphorylation activity (Lee et al., 2017). In addition, HDAC inhibitors increased ALDH1A1 expression through the transcription factor BRD4 in ovarian cancer cells (Yokoyama et al., 2016). ALDH1A2 played a role in conferring resistance to 13-cis-retinoic acid (13-cis-RA) in neuroblastoma cells (Hartomo et al., 2015). Also, the knockdown of ALDH1A3 markedly enhanced the sensitivity of lung adenocarcinoma to cisplatin (Yun et al., 2018). Together, these findings led us to investigate the expression of ALDHs as a metabolic resistance indicator. In this study, we found that the cisplatin-induced upregulation of ALDH1A1 was markedly enhanced by VPA in MDA-MB-231 cells but not in MDA-MB-436 cells (Figures 3A–F). As expected, this additivity was not seen in the H4 acetylation analysis (because cisplatin is not an HDAC inhibitor), implying different mechanisms involving the ALDH1A1 overexpression (Figures 3G–J). High ALDH1A1 levels further suggest that MDA-MB-231 cells could have acquired metabolic resistance. Based on these observations, we used the non-specific ALDH inhibitor, disulfiram, in our viability experiments, revealing that a combination treatment with disulfiram and VPA has significantly sensitized MDA-MB-231 cells to cisplatin, as compared with the other treatments (cisplatin, VPA-cisplatin, or disulfiram-cisplatin) (Figures 4A, B). Similar results were obtained in TNBC organoids (Figure 4D). In addition, we found that disulfiram combined with VPA–cisplatin can reprogram cellular metabolism by reducing the uptake of glucose, as compared to cisplatin with the other combinations (Figure 3C). Although disulfiram is a non-specific ALDH inhibitor, our findings provide support to the assumption that ALDH1A1 overexpression is associated with metabolic resistance and may represent an effective target for sensitizing cancer cells to cisplatin.

This study has limitations. First, we studied the drug effect on glucose metabolism *in vitro*, in only two TNBC cell lines (and an organoid model from a TNBC patient). Other cell lines representing different types of breast cancer, and *in vivo* studies, might be used for confirming our current findings. Second, we focused on cisplatin, with one paclitaxel study. It would be interesting to investigate the effect of VPA and disulfiram treatment on reprograming glucose metabolism in breast cancer cells following other chemotherapeutic treatments. Third, we did not specifically knockdown ALDH1A1 expression. Therefore, our results may indicate the effects of other ALDHs. An ALDH1A1 knockdown cell line should be constructed in future studies to investigate whether VPA treatment could result in cisplatin resistance in breast cancer cells; this might verify our present data in other aspects.

In summary, we demonstrated that VPA-cisplatin combination could differently reprogram the glucose metabolism of TNBC cells. The cisplatin-resistant MDA-MB-231 cells showed higher metabolic activity under VPA–cisplatin treatment. On the contrary, VPA had further decreased the oxidative phosphorylation rate that resulted in an early cell cycle of cisplatin-sensitive MDA-MB-436 cells. This effect might be attributed to the elevation of ALDH1A1 expression. Given the association of ALDH1A1 expression with the chemotherapeutic response in MDA-MB-231 TNBC cells, ALDH inhibitors which are capable of inhibiting ALDH1A1 may be a potential novel strategy for improving the chemotherapy response in TNBC patients, especially for the patients who are co-treated with VPA.

## Data Availability

The raw data supporting the conclusion of this article will be made available by the authors, without undue reservation.
