# Supplementary material for: Valproic acid reprograms the metabolic aberration of cisplatin treatment via ALDH modulation in triple-negative breast cancer cells
Source: Front Cell Dev Biol. 2023 Oct 26;11:1217149. doi: 10.3389/fcell.2023.1217149 (PMC10639136; doi:10.3389/fcell.2023.1217149)
Supplement: Supplementary file 1 [file Presentation1.PPTX]

## Slide 1
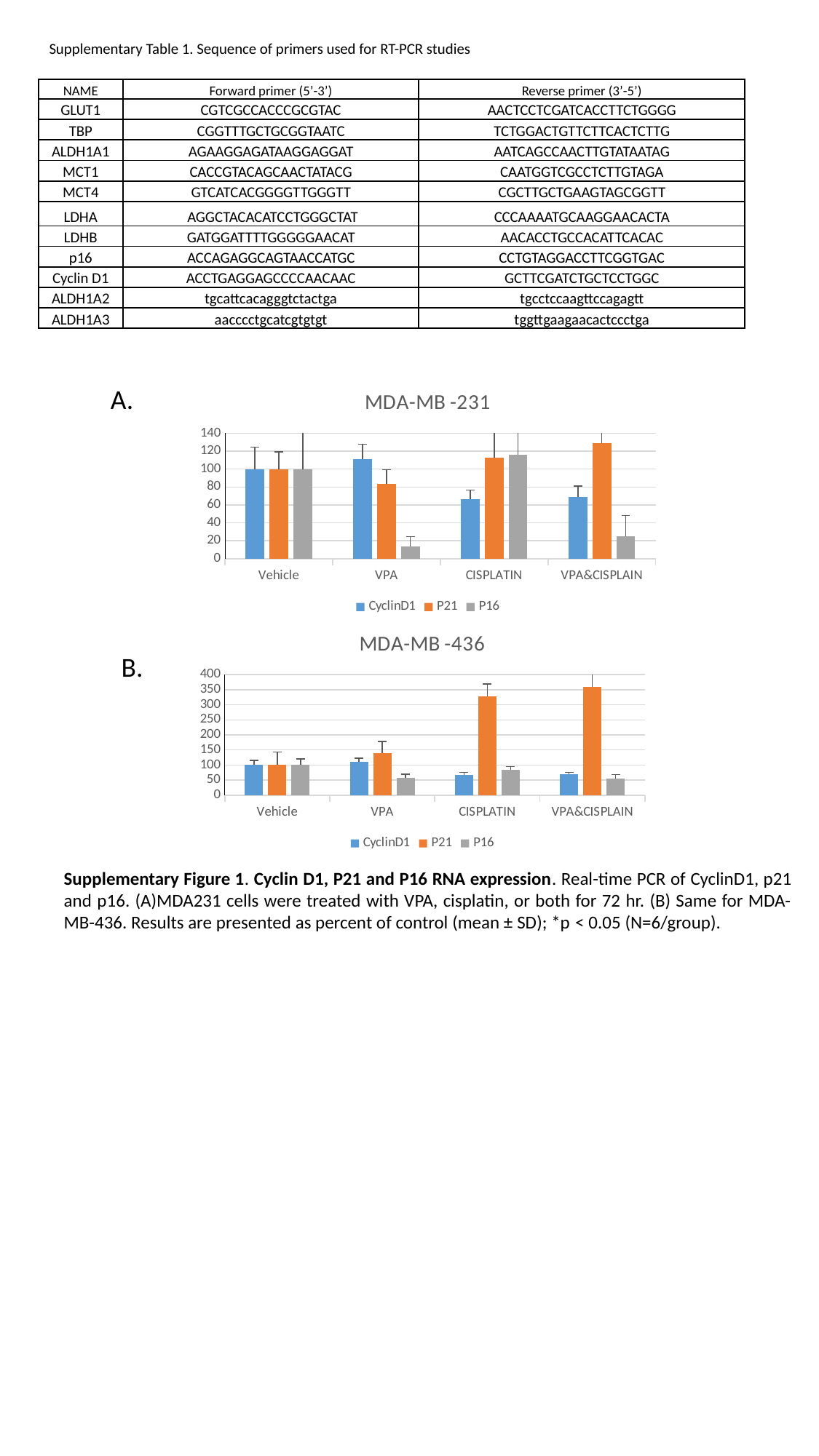

Supplementary Table 1. Sequence of primers used for RT-PCR studies
| NAME | Forward primer (5’-3’) | Reverse primer (3’-5’) |
| --- | --- | --- |
| GLUT1 | CGTCGCCACCCGCGTAC | AACTCCTCGATCACCTTCTGGGG |
| TBP | CGGTTTGCTGCGGTAATC | TCTGGACTGTTCTTCACTCTTG |
| ALDH1A1 | AGAAGGAGATAAGGAGGAT | AATCAGCCAACTTGTATAATAG |
| MCT1 | CACCGTACAGCAACTATACG | CAATGGTCGCCTCTTGTAGA |
| MCT4 | GTCATCACGGGGTTGGGTT | CGCTTGCTGAAGTAGCGGTT |
| LDHA | AGGCTACACATCCTGGGCTAT | CCCAAAATGCAAGGAACACTA |
| LDHB | GATGGATTTTGGGGGAACAT | AACACCTGCCACATTCACAC |
| p16 | ACCAGAGGCAGTAACCATGC | CCTGTAGGACCTTCGGTGAC |
| Cyclin D1 | ACCTGAGGAGCCCCAACAAC | GCTTCGATCTGCTCCTGGC |
| ALDH1A2 | tgcattcacagggtctactga | tgcctccaagttccagagtt |
| ALDH1A3 | aacccctgcatcgtgtgt | tggttgaagaacactccctga |
A.
### Chart: MDA-MB -231
| Category | CyclinD1 | P21 | P16 |
|---|---|---|---|
| Vehicle | 99.99999999999999 | 100.0 | 100.0 |
| VPA | 111.34848381489303 | 83.3213164862341 | 13.658742156714942 |
| CISPLATIN | 66.77139599023509 | 112.55196281200837 | 116.30441976958565 |
| VPA&CISPLAIN | 68.67672940279859 | 129.13335584151028 | 24.751551309561904 |
### Chart: MDA-MB -436
| Category | CyclinD1 | P21 | P16 |
|---|---|---|---|
| Vehicle | 99.99999999999999 | 100.0 | 100.0 |
| VPA | 111.34848381489303 | 140.68867171961855 | 56.544981647699366 |
| CISPLATIN | 66.77139599023509 | 327.7094942828356 | 84.73348412523274 |
| VPA&CISPLAIN | 68.67672940279859 | 360.60199188790756 | 54.72103908589583 |B.
Supplementary Figure 1. Cyclin D1, P21 and P16 RNA expression. Real-time PCR of CyclinD1, p21 and p16. (A)MDA231 cells were treated with VPA, cisplatin, or both for 72 hr. (B) Same for MDA-MB-436. Results are presented as percent of control (mean ± SD); *p < 0.05 (N=6/group).

## Slide 2
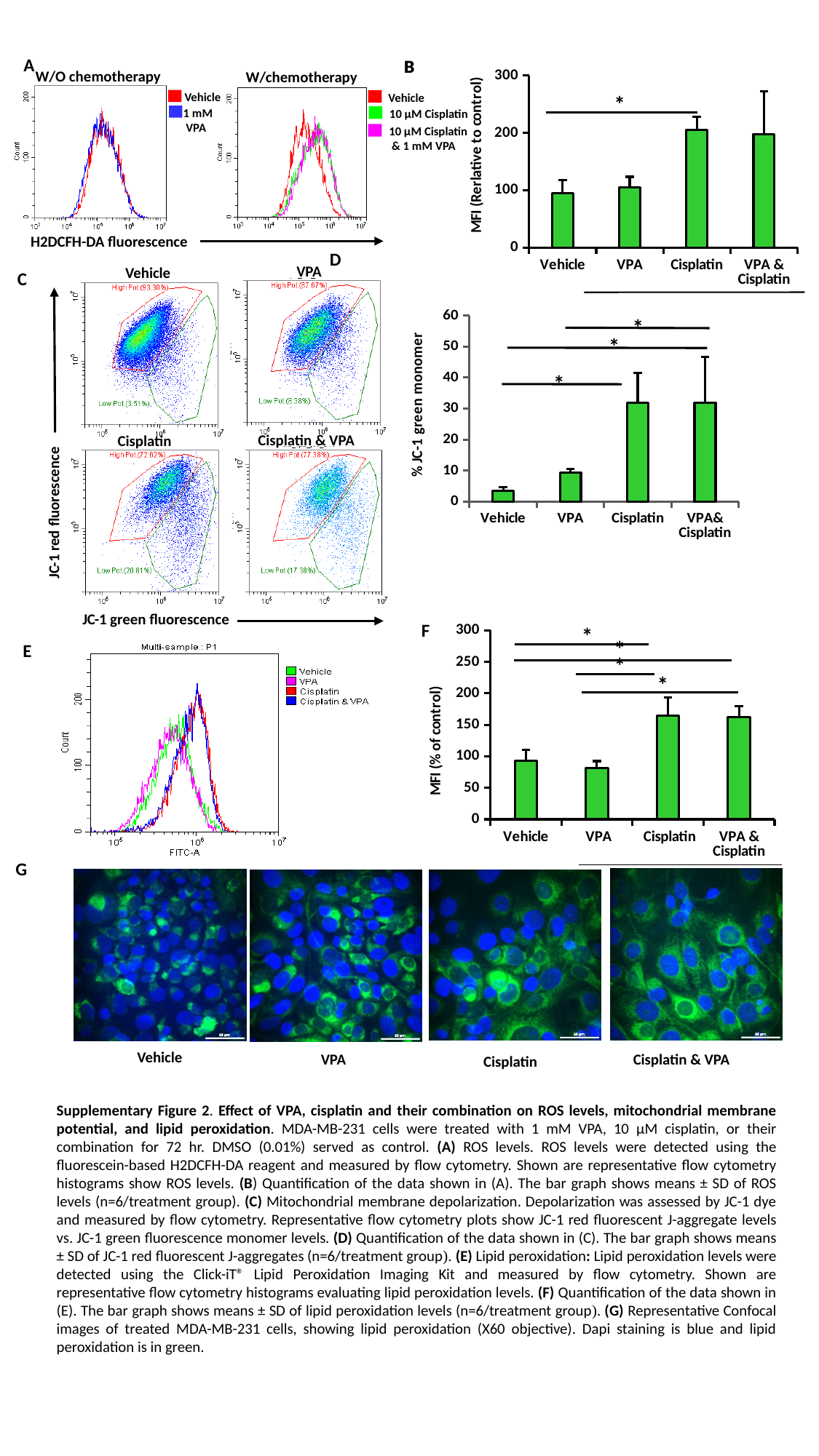

A
VPA
Vehicle
C
Cisplatin & VPA
Cisplatin
W/O chemotherapy
W/chemotherapy
B
B
### Chart
| Category | |
|---|---|
| Vehicle | 94.49566278931788 |
| VPA | 105.11664021709494 |
| Cisplatin | 204.61807838169747 |
| VPA & Cisplatin | 197.84608059449886 |Vehicle
1 mM
 VPA
Vehicle
10 µM Cisplatin
10 µM Cisplatin
 & 1 mM VPA
H2DCFH-DA fluorescence
D
### Chart
| Category | |
|---|---|
| Vehicle | 3.4579999999999997 |
| VPA | 9.343333333333332 |
| Cisplatin | 31.875 |
| VPA& Cisplatin | 31.828333333333333 |*
*
*
JC-1 red fluorescence
JC-1 green fluorescence
### Chart
| Category | |
|---|---|
| Vehicle | 92.56045434512293 |
| VPA | 81.67478091257469 |
| Cisplatin | 164.16439069422552 |
| VPA & Cisplatin | 161.91343438891707 |E
G
Vehicle
VPA
Cisplatin & VPA
Cisplatin
Supplementary Figure 2. Effect of VPA, cisplatin and their combination on ROS levels, mitochondrial membrane potential, and lipid peroxidation. MDA-MB-231 cells were treated with 1 mM VPA, 10 µM cisplatin, or their combination for 72 hr. DMSO (0.01%) served as control. (A) ROS levels. ROS levels were detected using the ﬂuorescein-based H2DCFH-DA reagent and measured by ﬂow cytometry. Shown are representative flow cytometry histograms show ROS levels. (B) Quantification of the data shown in (A). The bar graph shows means ± SD of ROS levels (n=6/treatment group). (C) Mitochondrial membrane depolarization. Depolarization was assessed by JC-1 dye and measured by ﬂow cytometry. Representative flow cytometry plots show JC-1 red fluorescent J-aggregate levels vs. JC-1 green fluorescence monomer levels. (D) Quantification of the data shown in (C). The bar graph shows means ± SD of JC-1 red fluorescent J-aggregates (n=6/treatment group). (E) Lipid peroxidation: Lipid peroxidation levels were detected using the Click-iT® Lipid Peroxidation Imaging Kit and measured by ﬂow cytometry. Shown are representative flow cytometry histograms evaluating lipid peroxidation levels. (F) Quantification of the data shown in (E). The bar graph shows means ± SD of lipid peroxidation levels (n=6/treatment group). (G) Representative Confocal images of treated MDA-MB-231 cells, showing lipid peroxidation (X60 objective). Dapi staining is blue and lipid peroxidation is in green.

## Slide 3
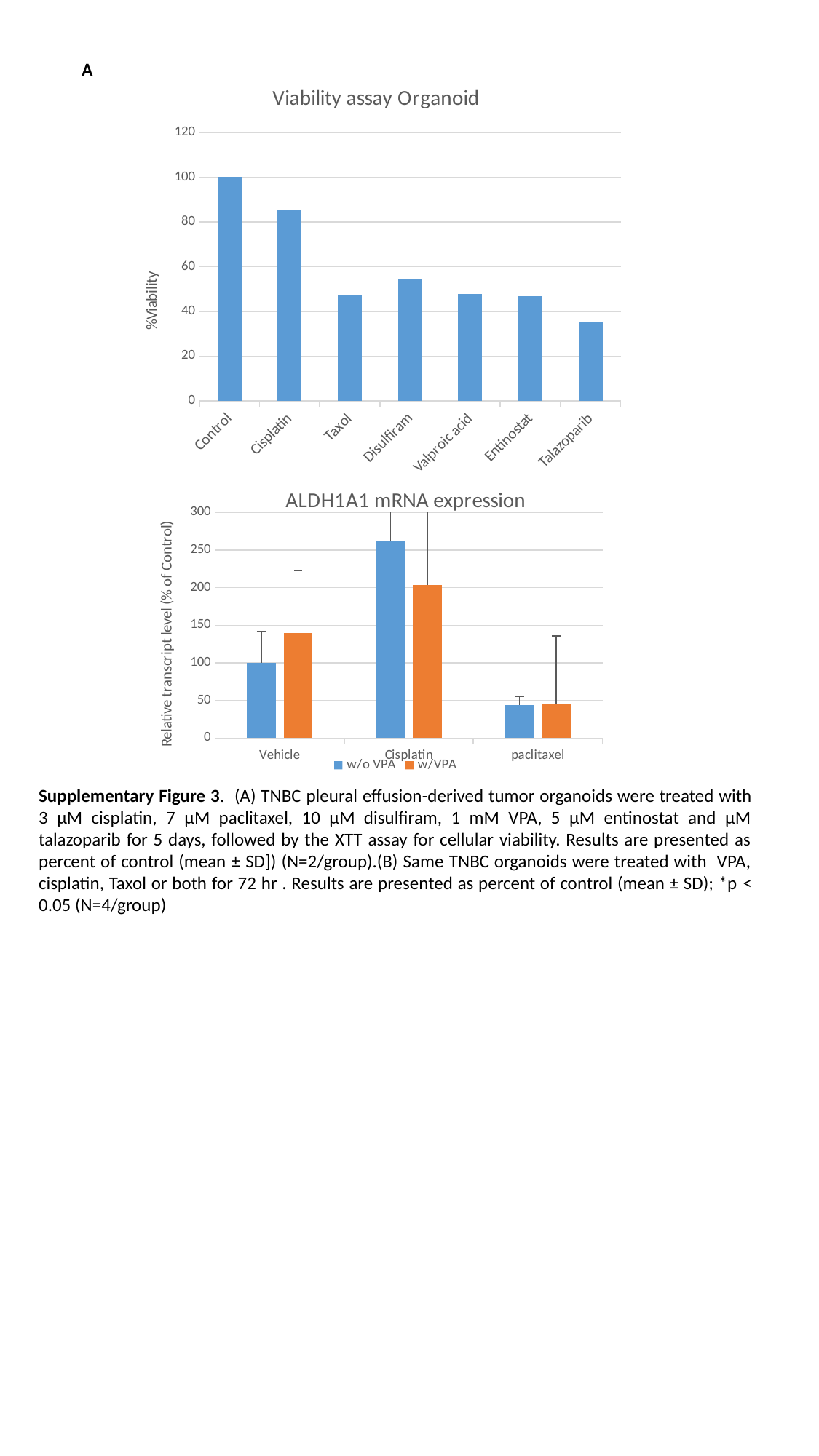

A
### Chart: Viability assay Organoid
| Category | |
|---|---|
| Control | 100.0 |
| Cisplatin | 85.48486720197654 |
| Taxol | 47.39551163269509 |
| Disulfiram | 54.64278361128269 |
| Valproic acid | 47.84846613135681 |
| Entinostat | 46.73666872555076 |
| Talazoparib | 34.91867407864938 |
### Chart: ALDH1A1 mRNA expression
| Category | | |
|---|---|---|
| Vehicle | 100.0 | 139.7899372300986 |
| Cisplatin | 261.44120513035153 | 203.3721527824198 |
| paclitaxel | 43.82054880337151 | 45.30525322702294 |Supplementary Figure 3. (A) TNBC pleural effusion-derived tumor organoids were treated with 3 µM cisplatin, 7 µM paclitaxel, 10 µM disulfiram, 1 mM VPA, 5 µM entinostat and µM talazoparib for 5 days, followed by the XTT assay for cellular viability. Results are presented as percent of control (mean ± SD]) (N=2/group).(B) Same TNBC organoids were treated with VPA, cisplatin, Taxol or both for 72 hr . Results are presented as percent of control (mean ± SD); *p < 0.05 (N=4/group)
